# Dialysed Iron

**Published:** 1878-12

**Authors:** 


### Dialysed Iron.

*Editors Lancet and Clinic.*—Having seen the happiest effects recently from the use of dialysed iron, administered to two female patients suffering from chlorosis, both of whom objected to taking iron in any form as it had always made them suffer more unpleasant effects than did the disease itself; and the rapid benefit following the administration of this comparatively new preparation of iron, led me to read up its chemistry and mode of preparation. In addition to the very favorable notice from Dr. S. Weir Mitchell, I was very much impressed with the article written by Dr. Yandell, of Louisville, and also by an analysis given in a late number of the Boston Medical and Surgical Journal, by Dr. Emory, of Boston. Messrs. Jno. Wyeth & Bro., of this city, having specially called the attention of the medical profession to this preparation, I took the liberty of calling upon them, and asked if they would give me an account of their mode of preparation and allow me to visit their laboratory and see the practical workings of their appliances for the manufacture of this iron. They at once expressed their willingness to give me every facility and asked me to visit the part of the building devoted to the manufacture of this article. Their very intelligent manager of this department explained to me fully the different processes from the crude iron to the bottled preparation. Thinking it might be of interest to the readers of your valuable journal I resolved to communicate with you upon the subject.

Instead of using a commercial iron in the form of iron wire and filings they use a chemically pure sulph. of iron. The entire freedom of the iron from any impurity is very essential. The pure sulphate of iron is precipitated in large vessels by means of ammonia. It is then carefully washed, drawn out, and drained into a large steam jacketed kettle, and mixed with the proper proportion of sesqui-oxide of iron and heated to a temperature of 160 degrees. This gives the proper solution of per-oxide of iron ready for the process of endosmosis.

The water they use to aid in the dialysation is furnished by an artesian well, dug for the purpose and the water is pumped into large vats on the roof of their four-story building.

The water in these tanks is heated by steam through coils of pipe, which are so arranged that cold water may be added so as to regulate the exact temperature as may be thought necessary for the proper dialysation—this temperature being varied as the per centage of acid is lessened in the solution.

Each appliance covers a surface of 400 square feet, enabling them to prepare about sixty gallons at one time with each one of their vessels. It requires from ten to thirty days to finish each separate acid solution placed upon the membrane. Every day during the process the solution is carefully assayed by the person in charge, so as to enable him to regulate the temperature of the water and prevent the membrane from being clogged by the iron solution. The essay is made by precipitating with aqua ammonia well washed. Heat is applied to expel the excess of ammonia in the solution. Nitrate of silver is added. The mixture is then allowed to stand and afterwards decanted, washed, dried and weighed. Washing, drying and weighing shows the percentage of iron in the solution.

The standard strength of the solution of iron is 24 grains to each fluid ounce of pure per-oxide of iron, each fluid ounce containing only sufficient chlorine to prevent decomposition. Occasionally if the dialysation is carried too far some portion of the solution will gelatinize from the dialyser, and occasionally if exposed to the sunlight or air too long before being bottled this solution of iron will become thick. If a small percentage of distilled water is immediately added it will regain its limpidity at once, but if allowed to remain in this condition for some time it undergoes exactly the same change that takes place with the officinal hydrated sesqui-oxide of iron when kept under water for a considerable time. This solution when properly prepared should be almost tasteless and yield no reaction of acid to litmus paper, or any of the ordinary tests.

The usual dose is from five to twenty drops given three or four times a day. Its freedom from taste renders it especially desirable for children. As experiment has shown that only a certain amount of iron will be absorbed into the system at one time, I cannot recognize the advantage of giving it in larger doses, although some medical men claim that they get better benefit when it is administered in half teaspoonful doses. Dr. Weir Mitchell especially advocates the larger doses. Dr. DaCosta and a number of our leading physicians seem to prefer smaller doses, usually from 10 to 20 drops as a full adult dose. The dose given to the patients to whom I make allusion above was 15 drops three times a day.

Physicians will readily understand why this solution of iron when properly prepared can be depended upon as an antidote for poisoning by arsenic. Its chemistry is almost identical with that of the hydrated sesqui-oxide of iron.

I was also much interested and instructed by the explanation they gave me concerning their method of percolation and manufacture of fluid extracts and resinoids.

At some future time, if you think it would prove interesting and instructive to your readers it would give me pleasure to give you a resumé of their mode of exhausting the drugs.

The very general attention now being given to sulphate of cinchona, cinchonidia, quinida, and the other less costly alkaloids existing in Peruvian bark; on account of the terrible high cost of quinine, is a subject of great interest to the profession. At some future time I will visit the extensive works of Messrs. Rosengarten and those of Messrs. Powers & Weightman and endeavor to learn some points in order that they may be laid before your readers.

